# Body mass index and atrial fibrillation recurrence post ablation: A systematic review and dose-response meta-analysis

**DOI:** 10.3389/fcvm.2022.999845

**Published:** 2023-02-02

**Authors:** Fuwei Liu, Tiangang Song, Qingwen Hu, Xin Zhu, Huilei Zhao, Ziqi Tan, Peng Yu, Jianyong Ma, Jun Luo, Xiao Liu

**Affiliations:** ^1^Department of Cardiology, The Affiliated Ganzhou Hospital of Nanchang University, Ganzhou, Jiangxi, China; ^2^Department of Endocrine, The Second Affiliated Hospital of Nanchang University, Nanchang, Jiangxi, China; ^3^Department of Cardiology, The First People’s Hospital of Yulin, Yulin, Guangxi, China; ^4^Department of Anesthesia, The Third People’s Hospital of Nanchang, Nanchang, Jiangxi, China; ^5^Department of Pharmacology and Systems Physiology, University of Cincinnati College of Medicine, Cincinnati, OH, United States

**Keywords:** body mass index, obesity, atrial fibrillation, exposure-effect, meta-analysis, radiofrequency ablation

## Abstract

**Objectives:**

The aim of this study was to evaluate the shape of the dose-response relationship between body mass index (BMI) and atrial fibrillation (AF) recurrence in patients who have undergone radiofrequency ablation.

**Methods:**

Studies investigating BMI and AF recurrence in patients with AF after ablation were identified through electronic searches in the PubMed, EMBASE, and Cochrane Library databases. The potential non-linear relationship was fitted using robust error meta-regression. Our study was registered with PROSPERO (CRD42019121373).

**Results:**

Twenty-six cohort studies with 7,878 cases/26,450 individuals were included, and a linear dose-response relationship between BMI and AF recurrence (*P*_non–linearity_ = 0.12) was found. The risk of AF recurrence in patients with a BMI over 28 was significantly increased. Specifically, for each 5 kg/m^2^ increase in BMI, the risk of AF recurrence increased by 15% (95% CI: 1.08–1.22) with moderate heterogeneity (*I*^2^ = 53%). Subgroup analyses showed that the pooled risk ratio was not significantly changed in subgroup analysis adjustment for the following important potential intermediate factors: left atrial diameter and obstructive sleep apnea.

**Conclusion:**

This study showed that there is a borderline positive linear association between BMI and AF recurrence post ablation. Overweight and obesity are significantly associated with AF recurrence.

**Systematic review registration:**

https://www.crd.york.ac.uk/PROSPERO/, identifier CRD42019128770.

## Introduction

Obesity is a rapidly growing global public health concern (1). For example, in the United States, nearly 65% of the population is overweight, and 31% is obese (2). The direct association between obesity and the incidence of atrial fibrillation (AF) has been well-established in large, long-term general population-based cohorts (3, 4). According to a report, obesity increased the risk of developing AF by 49% in the general population, and the risk increased in parallel with increasing body mass index (BMI) (5). AF is the most common cardiac rhythm dysfunction worldwide and leads to significant morbidity and mortality. Over the past decade, radiofrequency ablation, an alternative treatment for AF, has shown advantages over pharmacological methods of rhythm control and has evolved into an important therapy for AF. However, reports of long-term outcomes of ablation demonstrate reduced success over time. The actuarial recurrence at 2 years post ablation was 20% and increased to 40–45% at 5 years.

Body mass index, an important predictor of the incidence of AF in the general population, has also been linked to increased AF recurrence after ablation (6, 7). However, the results regarding the impact of BMI in patients undergoing ablation therapy have been controversial, with several studies demonstrating no clear association (7–11). Five meta-analyses have been conducted to show an increased risk of AF recurrence in patients with elevated BMI (12–16). These studies have provided valuable information; however, there are certain limitations to be addressed. First, a categorical model they applied has the risk of losing power and precision by dividing the exposure into several groups (17). Second, previous meta-analyses have not taken into account confounding factors between obesity and AF recurrence, and it remains unclear whether obesity independently increases the risk of AF recurrence. Second, previous meta-analyses have not taken into account confounding factors between obesity and AF recurrence, and it remains unclear whether obesity independently increases the risk of AF recurrence. Routine risk factors such as hypertension, diabetes, obstructive sleep apnea (OSA) and left atrial diameter (LAD) have been reported to be more prevalent in obese individuals and those undergoing ablation therapy. Also the 2020 ESC guidelines review OSA as one of the potential confounders between obesity and AF recurrence (18). Third, only one meta-analysis has examined dose-response analysis of BMI and AF recurrence after catheter ablation, but it included a limited number of articles and had high publication bias and heterogeneity. In addition, this study did not demonstrate a statistically significant effect of linear dose-response analysis on AF recurrence, and the effect estimate at the obesity threshold was not significant. Whether there are any threshold effects between BMI and the risk of AF recurrence after ablation is unclear, and clarifying the dose-response association is necessary and would be of major importance for facilitating better outcomes for patients with obesity and AF undergoing ablation. Thus, we conducted a meta-analysis to clarify the dose-response relationship between BMI and the risk of AF recurrence after ablation.

## Methods

The protocol of this study was registered with PROSPERO (International prospective register of systematic reviews) (https://www.crd.york.ac.uk/PROSPERO/: registration number-CRD 42019128770).

We performed this meta-analysis following the Preferred Reporting Items for Systematic Reviews and Meta-Analyses (PRISMA) statement (Supplementary Table 1).

### Literature search

Two authors (FL and XL) independently systematically searched the Cochrane Library, PubMed, and Embase databases for eligible studies until October 5, 2021. Three groups of keywords (linked to BMI, AF, radiofrequency ablation, respectively) were combined using the Boolean operator “and.” In addition, we searched the reference lists of three previous published meta-analyses (12, 13, 16) or other relevant publications to identify further studies. All discrepancies were resolved through discussion with each other. No language restrictions were applied in the whole literature search. The detail search strategy was provided in Supplementary Table 2.

### Study selection

According to the PICOS (population, intervention, comparison, outcome, and study design), the selection criteria were as follows: (i) population: patients with AF undergoing radiofrequency ablation; (ii) and exposure and comparison: high vs. low BMI level; (iii) outcomes: reported the association between of BMI on AF recurrence. No blank period was pre-defined: (iv) study design: cohort, nested case-control, or clinical trials. For multiple publications/reports created from the same data, the studies with the longest follow-up period or the largest number of AF cases were included. We only included studies with multivariate analysis. Case-control and univariate analysis studies were excluded considering their larger bias. Certain publication types (e.g., reviews, editorials, and animal studies), or studies with insufficient data were excluded from this study. The details reasons of excluded studies were listed in Supplementary Table 3.

### Data extraction and quality assessment

Basic information of each study, including the authors, publication year, region, study design, participants (sex, age), follow-up time, adjustments for confounders, categories of BMI and adjusted risk ratios (RRs) with its 95% confidence intervals (CIs) for each BMI category were extracted. If multi-adjusted RRs were reported in one study, we extracted the most completely adjusted one.

The quality of all included observational studies were assessed by using Newcastle-Ottawa quality assessment scale (NOS) (19). The validated NOS items with a total of 9 stars involved three aspects including the selection of studies, the comparability of cohorts, and the assessment of the outcome. A NOS score of ≥7 stars was considered as acceptable quality, otherwise, as low-quality studies (20).

### Statistical analyses

Summary RRs and 95% CIs for a 5-unit increment in BMI were using a random effects model. Study-specific slopes (linear trends) and 95% CIs from the natural logs of the reported RRs and CIs across categories of BMI were calculated by using the method of Greenland and Longnecker (21). Non-linear dose-response analysis were performed by using the robust error meta-regression method described by Xu and Sar (22). This method is based on a “one-stage approach” which treating each study as a cluster of the whole sample and considering the within study correlations by clustered robust error. It requires known levels of BMI and RRs with variance estimates for at least two quantitative exposure categories. For studies that did not set the lowest BMI group as a reference, data were transformed using a method described by Hamling et al. (23) which requires the number of cases and participants in each category. If these data could not be obtained from an article, the evidence was not pooled. If the median or mean BMI was not provided and reported in ranges, we estimated the midpoint of each category by averaging the lower and upper boundaries of that category (20). If the highest or lowest category was open-ended, we assumed that the open-ended interval length was the same as the adjacent interval. To assess the heterogeneity of RRs across studies, the *I*^2^ (95% CI) statistic was calculated with the following interpretation: low heterogeneity, defined as *I*^2^ < 50%; moderate heterogeneity, defined as *I*^2^ 50–75%; and high heterogeneity, defined as *I*^2^ > 75% (24, 25). Publication bias was explored by Egger’s test, Begg’s test, and funnel plot. Sensitivity analysis was performed by excluding a bank period < 3 months and leave one-out methods to confirm the robustness of primary analysis. *P*-value < 0.05 was considered statistically significant.

## Results

### Study selection

**FIGURE 1:**
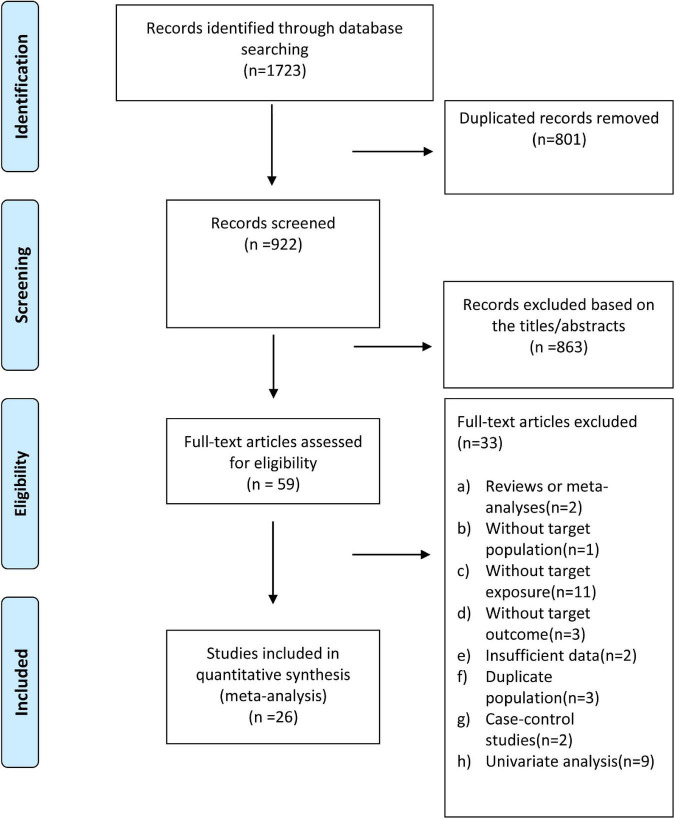
Flow diagram of study selection for the dose-response meta-analysis of body mass index and atrial fibrillation recurrence in patients undergoing radiofrequency ablation.

As shown in Figure 1, we identified 1,723 articles through an initial database search (Cochrane Library = 30, PubMed = 332, and EMBASE = 1,361) (Figure 1). After removing duplicate articles (*n* = 801), 922 studies remained. We further excluded 863 records by quickly screening the titles and abstracts, and 59 articles were reviewed in the detailed evaluation. Of the 59 records, 33 were excluded after the full-text review for the following reasons: (1) Reviews or meta-analyses (*n* = 2); (2) without target populations (*n* = 1); (3) without target exposure (*n* = 11); without target outcome (*n* = 3); (4) publications with insufficient data (*n* = 2); (5) studies based on duplicate population (*n* = 3); (6) case-control studies (*n* = 2); (7) univariate analysis (*n* = 9). Supplementary Table 3 provides the detailed reasons for exclusion following the full-text review. Finally, 26 (6–11, 26–45) studies were included in present study.

### Study characteristics and quality

Table 1 shows the baseline characteristics of the patients included in the study. Overall, the publication years ranged from 2008 to 2021, with ten conducted in North America (the USA and Canada), eight in Asia, and eight in Europe. The age range of patients in the included studies was 52–65 years, sample size range was 72–3,333, and follow-up time range was 3–42 months. Among the 26 articles, 14 and 12 was prospective and retrospective cohort studies, respectively. AF recurrence in the included studies was detected by using 12-lead ECG or 24 h Holter monitoring.

**TABLE 1:** Basic characteristics of the included studies in the dose-response meta-analysis of BMI and AF recurrence in patients post ablation.

References, country	Source of participants	AF cases, sample size	Mean age (years), male	Follow-up (month), blank period	Study design, AF detection	AF type		BMI data reported (kg/m^2^)	Adjustment for confounders	Ablation strategy
						**Par**	**Non-Par**			
Jongnarangsin et al. (26), USA	University of Michigan	127/324	57, 76%	12, 8 weeks	PC, ECG	72%	28%	<25 25–29 ≥30 Continuous	Age, gender, type and duration of AF, left atrial size, and LVEF	CPVI
Chang et al. (8), China	National Yang-Ming University School of Medicine	84/282	52, 76%	12, 2 months	PC, ECG	76.6%	23.4%	Continuous	HTN, diabetes, fasting glucose, triglycerides, high-density lipoprotein cholesterol, Mets score	CPVI
Letsas et al. (27), Germany	NA	28/72	55, 81%	13, 1 month	PC, ECG	64%	36%	Continuous	Use of statins, ACEIs/ARBs, spironolactone, WBC count, C-reactive protein	CPVI
Tang et al. (28), China	Beijing An Zhen Hospital	242/654	57, 71%	16, 3 months	RC, ECG	79.8%	20.2%	<25 ≥25 Continuous	AF duration, AF type, LAD, left ventricular end-diastolic diameter and ablative strategy	CVPI
Chilukuri et al. (10), USA	Johns Hopkins Hospital	34/109	60, 78%	11, 3 months	PC, ECG	67%	33%	<25.0 25.0–29.9 ≥30.0 Continuous	Age, persistent AF, OSA, female, left atrial size, LVEF	SPVI
Patel et al. (6), USA	Medical Center, Cleveland	781/3265	56, 84%	12, 8 weeks	RC, ECG	53.3%	46.7%	<30 ≥30	Age, type of AF, echo parameters, type II DM, HTN, CAD	PVAI
Kang et al. (7), Korea	Korea University Anam Hospital	32/94	59, 81%	9, 3 months	PC, ECG	NA	NA	Continuous	AF duration, DM, and required cardioversion during ablation	CPVI
Cai et al. (29), China	Hospital of Chongqing Medical University	47/186	55, 66%	24, 3 months	PC, ECG	85.6%	13.4%	<25 ≥25	MetS, AF type, duration of AF history, LAD, DM, ablation strategies, procedural failure, and ERAF.	SPVI or CPVA
He et al. (30), China	Beijing Anzhen Hospital	106/330	59, 68%	12, 3 months	RC, ECG	100%	0%	Continuous	Gender, HTN, Hs-CRP, serum triglyceride, LAD, and eGFR	CPVI
Sotomi et al. (31), Japan	Sakurabashi-Watanabe Hospital	40/392	62, 76%	32, 3 months	RC, ECG	71.2%	28.8%	<25 ≥25	HTN, persistent AF, high CRP	CVPI
Baek et al. (32), Japan	Yonsei AF ablation cohort in Japan	523/1,825	58, 74%	42, 3 months	PC, ECG	NA	NA	<25 ≥25	Age, sex, persistent AF, dyslipidemia, and HTN	CPVI
Bunch et al. (33), USA	LDS Hospital or Intermountain Medical Center	1,067/1,558	65, 61%	36, 3-month	RC, ECG	56.9%	43.1%	21–25 26–30 >30	Age, sex, HTN, hyperlipidemia, DM, HF, renal failure, OSA, prior cardioversion, CHADS2	PVI
Winkle et al. (9), USA	Sequoia Hospital	678/2,715	64, 70%	12, 3-month	PC, ECG	32.7%	54.9%	<35 ≥35	Age, LAD, sex, AF type, previous cardioversion, number of AADs failed, OSA, previous CVA/TIA, diabetes, CAD, AF duration	CPVI
Sivasambu et al. (35), USA	Johns Hopkins Hospital	354/701	59, 72%	12, 3-month	RC, ECG	59.1%	40.9%	18.5–25 25–30 30–40 >40	HTN, OSA, CHA2DS2-VASC score, and persistent AF	PVI
Glover et al. (36), Canada	Cardiology of Queen’s University	636/3,333	58, 68%	20, 3 month	PC, ECG	67.2%	27.8%	<25 25–30 >30 Continuous	AAD after procedure, achievement of exit and entrance block, first or redo procedure, HTN, CHA2D2S-VAS, OSA, type of AF	CPVI or SPVI
Providencia et al. (37), multi-country in Europe	7 European centers	916/2497	61, 72%	12, 3-month	PC, ECG	57.6%	32.8%	Continuous	Age, female, AF duration, AF type, mean of procedures time, CHA2DAS2-VASC, CHF, HTN, DM, stroke, TIA, vascular disease, OSA, eGFR, LAD, LVEF, procedure duration, additional LA line, AAD, fluoroscopy duration, cavotricuspid isthmus ablation	PVI
Trines et al. (38), Sweden	ESC-EHRA-AFA LT registry	1008/3069	59, 68%	12, 3 months	PC, mixed	68.3%	27%	Continuous	HTN	CPVI
Baek et al. (39), Korea	Inha University College of Medicine and Inha University Hospital	416/2221	55, 79%	54, 3 months	RC, ECG	59%	NA	<25 ≥25	Age, sex, non-paroxysmal AF, HF, HTN, DM, LAD, AF duration, and MR LGE ≥ 25%	CPVI
Bose et al. (40), USA	NA	19/103	60, 29%	12, NA	RC, ECG	100%	0	<25 25–30 30–35 35–40 >40	Age, sex, CAD, CHD, HTN, DM, dyslipidemia, valvular heart diseases, beta blocker, CCB, AAD	CPVI
Deng et al. (11), China	Guangdong General Hospital	365/1,410	57, 68%	21, 3 months	PC, ECG	NA	NA	18.5–25 ≥30 Continuous	Age, gender, bundle branch block, AF duration, COPD, alcohol consumption, smoking, HTN, DM, stroke/TIA, CAD, LVEF, vascular disease, early recurrence, LAD, and AF type	CPVI
De Maat et al. (34), Netherlands	University Medical Center Groningen	146/414	56, 76%	46, 3-month	RC, ECG	75%	25%	Continuous	Age, sex, OSA, AAD, LAD, AF duration, AF type, CHD, total cholesterol, number of PVI procedures HTN, DM, vascular disease, and stroke	CPVI
Donnellan et al. (42), USA	Cleveland Clinic	87/267	65, 69%	12, 3 months	RC, ECG	NA	NA	<31.7 ≥31.7	OSA, AF type, and LA size, OSA, HF	CPVI
Kong et al. (43), USA	University of Chicago	217	64, 68.2%	12, NA	RC, ECG	Na	Na	<30 ≥30	Age, gender, smoking, HTN, DM, HF, eGFR, and type of AF	PVI
Calero Nunez et al. (41), Spain	Albacete University Hospital	34/114	54, 70%	12, NA	RC, ECG	70.2%	29.8%	Continuous	Type of AF, OSA, LA size, ejection fraction, and HTN	CPVI
Abou El Khair (44), Sweden	Region Värmland and Örebro	19/90	63, 73%	12, 3 months	RC, ECG	NA	NA	Continuous	Age, sex, CHA2DS2-VASc score, DM, HF, HTN, LVEDd, and right atrial area.	CPVI
Mugnai et al. (45), Belgium	Electrophysiology centre of Brussels	89/208	59, 66%	62, 3 months	RC, ECG	100%	0	Continuous	Duration of symptoms, LAD	Index PV isolation

RC, retrospective cohort; PC, prospective cohort; ECG, electrocardiograph; NA, not available; PVI, pulmonary vein isolation; CPVI, circumferential pulmonary vein isolation; SPVI, segmental pulmonary vein isolation; CPVA, circumferential pulmonary vein ablation; LAD, left atrial diameter; MetS, metabolic syndrome; ERAF, early recurrence of AF after ablation; OSA, obstructive sleep apnea; WBC, white blood cell; CRP, C-reactive protein; CAD, coronary artery disease; AAD, antiarrhythmic drugs; CHRD, centre for heart rhythm disorders; LVEF, left ventricular ejection fraction; eGFR, estimated glomerular filtration rate; ACEIs/ARBs, ACE inhibitors/ARB inhibitors; SHD, structural heart disease; CHF, congestive HF; CVA, cerebrovascular accident; TIA, transient ischemic attack; AF, atrial fibrillation; BMI, body mass index; Par, paroxysmal; HTN, hypertension; COPD, chronic obstructive pulmonary disease; USA, United States of America; MR LGE, late gadolinium enhancement on cardiac magnetic resonance.

The quality of the articles was acceptable, with a majority (*n* = 23) of studies scores Newcastle-Ottawa Scale ≥ 7 points (Supplementary Table 4).

### Dose-response analysis of the association of BMI with the risk of post ablation AF recurrence

**FIGURE 2:**
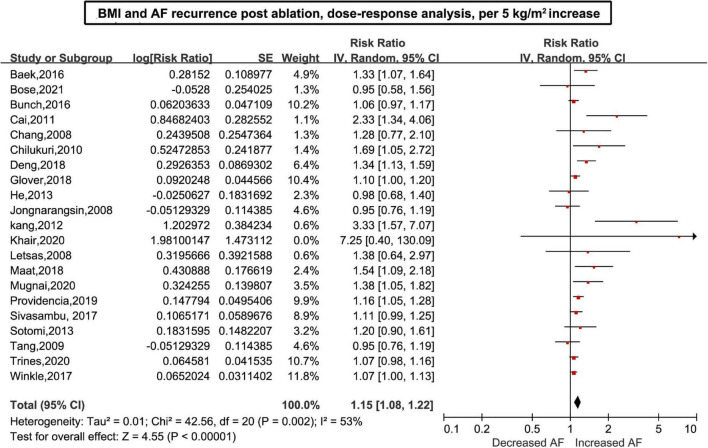
Body mass index and atrial fibrillation recurrence in patients undergoing radiofrequency ablation based on a dose-response analysis, per 5 units.

**FIGURE 3:**
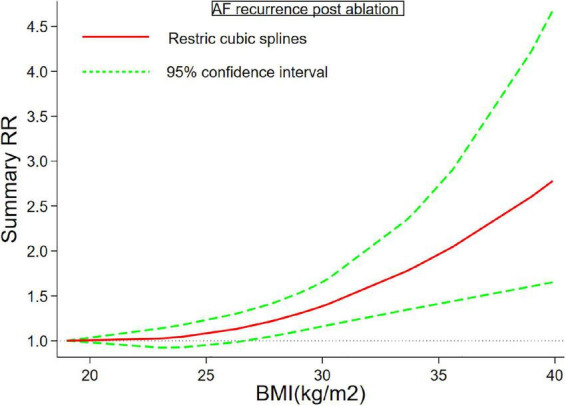
Body mass index and atrial fibrillation recurrence in patients undergoing radiofrequency ablation based on a non-linear dose-response analysis. The solid and dashed lines represent the estimated relative risk and the 95% confidence interval, respectively.

Twenty-six (6–11, 26–45) studies including 7,878 cases/26,450 individuals were included in the dose-response analysis. The summary RR for a 5-unit increase in BMI was 1.15 (95% CI: 1.08–1.22, *I*^2^ = 53%), with moderate heterogeneity (Figure 2). There was little evidence of heterogeneity (*I*^2^ = 34%) after excluding studies from Cai et al. (29) and Kang et al. (7), but a positive association remained (RR = 1.12, 95% CI: 1.07–1.18). In the non-linear analysis, we found a borderline positive linear association between BMI and AF recurrence after ablation (*P*_non–linearity_ = 0.12) (Figure 3). Supplementary Table 5 displays the RR estimates and 95% CIs from the specific BMI values derived from the dose-response figures.

### Subgroup analysis, sensitivity analysis, and publication bias

There may be a stronger association between BMI and AF recurrence among groups with larger AF recurrence cases (*P*_heterogeneity_ = 0.006). The positive association between BMI and AF recurrence persisted in almost all subgroups stratified by age, time of follow-up, region, and sample size after adjusting for confoundings (e.g., OSA), and there was little evidence of heterogeneity across these subgroups (Table 2).

**TABLE 2:** Subgroup analysis of BMI and AF recurrence in patients after catheter ablation, dose-response analysis, per 5 unit increase.

Items		Number of studies	RR	95% CI	*I* ^2^	*P*	
						Within subgroup	Between subgroup
**Result of primary analysis**		**21**	**1.15**	**1.08, 1.22**	**54%**	0.002	**NA**
Age*	≤60	13	1.10	1.03, 1.17	38%	0.005	0.19
	>60	7	1.20	1.08, 1.33	60%	0.003	
Study design	Retrospective cohort	10	1.11	1.02, 1.21	39	0.001	0.12
	Prospective cohort	11	1.25	1.11, 1.39	68	0.001	
Region	Northern America	8	1.08	1.03, 1.12	0%	0.0003	0.06
	Europe	6	1.18	1.06, 1.33	46%	0.004	
	Asia	7	1.30	1.08, 1.56	65%	0.006	
NOS scores	<7 scores	2	1.16	1.00, 1.36	26%	0.06	0.76
	7 or high scores	19	1.13	1.07, 1.20	51%	<0.001	
Publication years	2008–2013	9	1.13	1.07, 1.19	43%	<0.001	0.28
	2014–2021	12	1.28	1.03, 1.60	65%	0.03	
Sample size	≤1,000	14	1.24	1.08, 1.43	57%	0.003	0.11
	>1,000	7	1.10	1.05, 1.15	22%	0.0001	
Cases	≤100	9	1.70	1.26, 2.31	54%	0.006	0.006
	>100	12	1.10	1.05, 1.14	26%	0.001	
Adjusted factors	Age (+)	11	1.21	1.04, 1.21	57%	0.003	0.26
	Age (−)	10	1.23	1.07, 1.42	60%	0.004	
	Sex (+)	13	1.19	1.09, 1.31	59%	<0.001	0.90
	Sex (−)	8	1.21	1.05, 1.40	63%	0.01	
	DM (+)	11	1.29	1.14, 1.47	76%	<0.001	0.02
	DM (−)	10	1.09	1.02, 1.17	11%	0.001	
	left atrial size (+)	9	1.16	1.04, 1.30	64%	0.004	0.86
	left atrial size (−)	12	1.15	1.06, 1.23	45%	0.04	
	Hypertension (+)	15	1.14	1.08, 1.20	30%	<0.001	0.37
	Hypertension (−)	6	1.26	1.02, 1.57	73%	0.03	
	AF categories (+)	10	1.11	1.04, 1.18	42%	0.08	0.07
	AF categories (−)	11	1.29	1.11, 1.51	62%	0.001	
	Obstructive sleep apnea (+)	7	1.11	1.05, 1.17	36%	<0.001	0.23
	Obstructive sleep apnea (−)	14	1.20	1.07, 1.36	60%	0.003	

AF, atrial fibrillation; BMI, body mass index; DM, diabetes mellitus. *The mean age of Khair et al. was not available. Bolded values are meant to be the total results of all studies before subgroup analysis.

In the sensitivity analysis, the pooled results were stable when omitting one study at a time, with an RR range from 1.13 (95% CI: 1.07–1.20, *I*^2^ = 47%) to 1.16 (95% CI: 1.09–1.25, *I*^2^ = 53%). The result was also stable by excluding studies with a blank period < 3 months (RR: 1.15, 95% CI: 1.08–1.23) (Supplementary Figure 1). Publication bias was assessed using Egger’s test (*p* = 0.004), Begg’s test (*p* = 0.019), or funnel plots (Supplementary Figure 2). However, metatrim analysis showed that the addition of six missing studies did not significantly change the results, with a decreased magnitude of the pooled effect size (RR: 1.11; 95% CI: 1.03–1.18) (Supplementary Figure 3).

## Discussion

### Main findings

Based on available cohort studies, we showed a borderline positive linear dose-response relationship between baseline BMI and AF recurrence in patients after ablation. We found a 15% increase in AF recurrence per 5-unit increase in BMI. These results were confirmed in all subgroups stratified by age, sex, study design, region, follow-up duration, sample size, and adjustment. Overall, our results showed that overweigh (BMI > 28 kg/m^2^) or obesity was independently positively associated with AF recurrence after ablation.

Several well-known risk factors, including age, hypertension, diabetes, and enlarged LAD, have been linked to the recurrence of AF after ablation. Moreover, these comorbidities were common in patients with obesity. Therefore, whether the role of obesity is independent of these comorbidities is still unclear. For example, Jongnarangsin et al. showed that obesity was not associated AF recurrence post AF ablation after adjustments (26), suggesting obesity may simply be a phenotypic marker of profibrillatory comorbidities and not the key predictor of ablation failure. Moreover, although several previous meta-analyses (12, 13, 16) showed that obesity was associated with an increased risk of AF, none of them conducted a stratified analysis according to these confounding factors; therefore, this relationship is still under debate. We showed that the positive association of BMI and AF recurrence after ablation was still positive in all subgroups after adjustments, including those for hypertension, diabetes, age, OSA, and LAD (Table 2), which suggested that obesity was a factor contributing to AF recurrence, independent of conventional risk factors.

Although we found a linear association between BMI and AF (*p* for non-linearity = 0.12), we should explain these results with caution. As shown in Figure 3, we should figure out that the dose-response curve is much steeper at the BMI > 35. Specifically, a change in BMI of 25–30 kg/m^2^ increases the RR from 1.08 to 1.38, while RR increased sharply (1.95–2.78) between the 35 and 40 kg/m^2^ BMI group, somewhat reflecting a non-linear trend at higher BMI values. This observation reinforces the previous findings, showing severe obesity was a strong risk factor for cardiovascular diseases, including incident AF. A previous meta-analysis (14) also examined the dose-response analysis of BMI and risk of AF recurrence, but it failed to demonstrate the existence of a linear dose-response relationship. Although we demonstrated a borderline linear relationship between BMI and AF recurrence, the study demonstrated a dramatic increase in the risk of AF recurrence when BMI was greater than 35 kg/m^2^, so we would carefully interpret the borderline linear association. We should include more relevant studies. The non-linear p value might be significant if more population with high BMI were include. Overall, we should point out that grade II obesity substantially increased the risk of AF relapse after ablation. Weight loss intervention might be encouraged for these patients with severe obesity to reduce rate of AF relapse. 2020 European Society of Cardiology guidelines did not set a BMI threshold value for weight loss in obese patients who plan to receive AF ablation, while our results provide a valuable information for this.

### Comparison with other studies

In the context of AF ablation, obesity was not a new topic. Several reviews have shown that obesity increases the risk of AF relapse after ablation. Three showed that the obese group had a significantly increased risk of AF recurrence (13, 15, 16). In another excellent review by Wong et al., for every 5-unit increase in BMI, there was a 13% greater excess risk of post AF ablation (12). A recent meta-analysis included 12 studies reported a positive non-linear dose-response relationship between BMI and AF ablation (14). Interestingly, with the inclusion of more studies (*N* = 26), our results were opposite to theirs, showing a borderline positive linear dose-response relationship (*P* = 0.16), revealing a threshold effect between BMI and the risk of AF recurrence after ablation. In addition, the five published meta-analyses did not take into account confounding factors such as age, hypertension, AF category, diabetes, OSA, and LAD and did not demonstrate whether obesity could independently increase the risk of AF recurrence. We showed that the positive association of BMI and AF recurrence after ablation was still positive in all subgroups after adjustments, which suggested that obesity was a factor contributing to AF recurrence, independent of conventional risk factors.

### Underlying mechanism

Several potential mechanisms could explain this association. First, obesity could result in many changes involving various domains, such as hemodynamics, neurohumoral factors, inflammatory factors, metabolic factors, adipokines, and autonomics. For example, obesity leads to a high cardiac output state and the presence of left ventricular hypertrophy (eccentric or concentric) in association with left ventricular diastolic dysfunction (46). The hemodynamic changes associated with the elevation in left atrial and systolic blood pressure and left ventricular diastolic dysfunction contribute to atrial stretch and “trigger” AF (47). Furthermore, obesity is considered a state of chronic low-grade inflammation (48, 49). Studies have shown that patients with a higher BMI exhibit increased levels of several inflammatory and oxidative stress markers, including high-sensitivity C-reactive protein, serum creatinine, fibrinogen, and uric acid (50). Numerous studies have also demonstrated the implication of inflammation and oxidative stress in the pathophysiology of AF (51, 52). Therefore, it is reasonable to assume that obesity-associated inflammation may contribute to AF recurrence after ablation. Finally, it must be noted that the pathophysiological mechanisms linking obesity and AF are highly complex and remain incompletely understood. It is likely that a combination of multiple factors previously mentioned contributes to the recurrence of AF after ablation.

### Future research

Some authors proposed that obesity was a phenotypic marker of OSA that is associated with AF recurrence. Obesity is an important risk factor for the incidence of OSA (53), and the association between OSA and AF has been explored extensively (28). A meta-analysis reported that patients with OSA had a 25% greater risk of AF recurrence after ablation than those without OSA (53). The present study showed that the positive association was significant regardless of adjustment for OSA, which suggested that obesity increased the risk of AF recurrence independently of OSA. Therefore, we hypothesized that OSA may not be a mechanism by which obesity results in AF recurrence. Furthermore, a large retrospective cohort study also showed that obesity remains a powerful predictor of incident AF in patients without OSA based on the gold standard diagnostic test (54). This finding was consistent with our results, which suggested that obesity contributes to AF recurrence after ablation independent of OSA. However, considering the limited studies in the subgroup (*N* = 7), further prospective studies are needed to confirm our results.

The association between underweight and AF relapse is still inconclusive. Based on a retrospective cohort study in China, Deng et al. showed that underweight (BMI < 18.5 kg/m^2^) significantly increased the risk of AF recurrence (HR 1.85, *P* = 0.02) (11). In contrast, by using a different definition of underweight (BMI < 20 kg/m^2^), Bunch et al. did not find an elevated risk of AF recurrence of underweight in the US (33). However, the scarcity of studies precludes us from performing a dose-response analysis, which may be attributed to the high prevalence of obesity in Western countries. However, evidence from several population-based studies has shown that underweight is a risk factor for AF. Therefore, although the results regarding underweight and AF relapse were inconclusive, we supposed there may be a positive association between underweight and the risk of AF recurrence in patients undergoing ablation. More studies are warranted to address this issue.

In addition, although BMI is widely adopted because it is convenient to apply and inexpensive, BMI is not perfect as a measure of obesity. Because BMI is an indirect measure of obesity, it cannot distinguish between fat and lean tissue and can produce a certain amount of error (55). The problem may lead to misclassification of the experimental population in terms of body fat and may introduce bias. The impact of other parameters of body fat and body composition (e.g., waist circumference and waist-hip ratio) on outcomes among patients undergoing ablation should be further assessed by future studies.

### Policy implications

As shown in our results, the likelihood of AF recurrence after ablation increasing with BMI. In patients with grade II obesity (BMI > 35 kg/m^2^), the risk of AF recurrence increased by 95%. Our findings provide valuable insight for the clinical prevention of AF recurrence after ablation. For patients with severe obesity (BMI > 35 kg/m^2^), cardiologists should consider that their likelihood of AF relapse is much higher than that of individuals with a normal BMI. Therefore, for those patients strongly considering AF ablation, the risks and benefits should be carefully evaluated.

Weight loss is associated with improvements in risk factors (e.g., hypertension, OSA, and glycemic control), decreased morbidity and mortality, and a decreased risk of AF. Evidence from observational studies, such as the Long-Term Effect of Goal-Directed Weight Management in an Atrial Fibrillation (LEGACY) study, showed that long-term weight loss (bariatric surgery or weight management) could reduce AF risk and burden in people with AF (56). Moreover, the benefit of weight loss from the prevention of AF was dose-dependent. A 10% weight loss with accompanied risk factor modification reduced the risk among arrhythmia-free patients by 45% compared with that associated with 3% weight loss (56). Therefore, current guidelines set the weight loss target at > 10% weight reduction with lifestyle modifications for BMI < 27 kg/m^2^ (18) to improve AF ablation. However, weight loss, either through lifestyle or bariatric surgery, is typically a long process. It is well-described that increasing the duration of AF has negative effects on ablation success rates. The time required for weight loss would have resulted in a delay of ablation of at least 6 months, which would have led to AF progression with worsening of success rates. Therefore, it is not recommended for all patients with overweight or obesity to lose weight; the benefits and risks should be carefully considered.

### Strengths and limitations

The strengths and limitations of this study merit careful consideration. This is the first dose-response meta-analysis that demonstrated a positive linear association between BMI and AF recurrence after ablation with a large sample size and robustness of the findings based on multiple subgroups (e.g., age, sex, duration of follow-up and adjustment for confounding and potential intermediate factors). However, it was limited by the inclusion of observational studies, which cannot definitively prove causation. Although most studies were adjusted for other comorbidities, it is impossible to fully take into account all confounding factors. For example, the dose of antiarrhythmic medication may differ between studies, and studies have shown that LA volume is more accurate than diameter. Second, several studies have shown that underweight and AF patients have worse outcomes (11). However, due to data restrictions, the impact of underweight (BMI < 18.5) on AF recurrence after ablation was not analyzed and needs to be further investigated. Third, BMI may not accurately indicate the degree of body adiposity, and the impact of other parameters of body fat and body composition (e.g., waist circumference and waist-hip ratio) on outcomes among patients undergoing ablation should be further assessed by future studies. Fourth, ablation techniques across studies may also influence our results. Overall, further well-designed, larger clinical trials are needed to confirm our results.

## Conclusion

This study demonstrated a positive independent association between BMI and AF recurrence among patients undergoing ablation, and overweight and obesity were found to be significantly associated with AF recurrence. Further well-designed, prospective studies are required to determine the effect of weight loss on AF recurrence post ablation.

## Data availability statement

The original contributions presented in this study are included in this article/Supplementary material, further inquiries can be directed to the corresponding author.

## Author contributions

JL was responsible for the entire project and revised the draft. FL, TS, and XL performed the systematic literature review and drafted the first version of the manuscript. All authors participated in the interpretation of the results and prepared the final version of the manuscript.
