# Supplementary material for: Body mass index and atrial fibrillation recurrence post ablation: A systematic review and dose-response meta-analysis
Source: Front Cardiovasc Med. 2023 Feb 2;9:999845. doi: 10.3389/fcvm.2022.999845 (PMC9932032; doi:10.3389/fcvm.2022.999845)
Supplement: Supplementary file 1 [file Data_Sheet_1.docx]

# Body mass index and atrial fibrillation recurrence in patients post ablation-A systematic review and dose-response meta-analysis

Menglu Liu1*, M.D., Fuwei Liu2*, M.D., Kaibo Mei3,M.D., Peng Yu4, M.D., Jianyong Ma5, M.D.,Yujie Zhao1, M.D., Wengen Zhu6, M.D., Xiao Liu7,8, M.D.

1 Department of Cardiology, Seventh People's Hospital of Zhengzhou, Zhengzhou, Henan, China

2 Department of Cardiology, the Affiliated Ganzhou Hospital of Nanchang University, Jiangxi, China

3 Department of Anesthesia, the People’s Hospital of Shangrao, Jiangxi, China

4 Department of Endocrine, the Second Affiliated Hospital of Nanchang University, Nanchang, Jiangxi, China

5 Department of Pharmacology and Systems Physiology, University of Cincinnati College of Medicine, Cincinnati, USA

6 Department of Cardiology, the First Hospital of Sun Yat-sen University, Guangzhou, Guangdong, China

7 Department of Cardiology, Sun Yat-sen Memorial Hospital of Sun Yat-sen University, Guangdong, China
8 Guangdong Province Key Laboratory of Arrhythmia and Electrophysiology, Guangdong, China

**SUPPLEMENTAL TABLES**

**Table S1:** PRISMA 2009 Checklist

| **Section/topic** | | **#** | | **Checklist item** | | **Reported on page #** | |
| --- | --- | --- | --- | --- | --- | --- | --- |
| **TITLE** | | | | | |  | |
| Title | | 1 | | Identify the report as a systematic review, meta-analysis, or both. | | 1 | |
| **ABSTRACT** | | | | | |  | |
| Structured summary | | 2 | | Provide a structured summary including, as applicable: background; objectives; data sources; study eligibility criteria, participants, and interventions; study appraisal and synthesis methods; results; limitations; conclusions and implications of key findings; systematic review registration number. | | 1-2 | |
| **INTRODUCTION** | | | | | |  | |
| Rationale | | 3 | | Describe the rationale for the review in the context of what is already known. | | 3 | |
| Objectives | | 4 | | Provide an explicit statement of questions being addressed with reference to participants, interventions, comparisons, outcomes, and study design (PICOS). | | 3 | |
| **METHODS** | | | | | |  | |
| Protocol and registration | | 5 | | Indicate if a review protocol exists, if and where it can be accessed (e.g., Web address), and, if available, provide registration information including registration number. | | 4 | |
| Eligibility criteria | | 6 | | Specify study characteristics (e.g., PICOS, length of follow-up) and report characteristics (e.g., years considered, language, publication status) used as criteria for eligibility, giving rationale. | | 4 | |
| Information sources | | 7 | | Describe all information sources (e.g., databases with dates of coverage, contact with study authors to identify additional studies) in the search and date last searched. | | 4 | |
| Search | | 8 | | Present full electronic search strategy for at least one database, including any limits used, such that it could be repeated. | | 4 | |
| Study selection | | 9 | | State the process for selecting studies (i.e., screening, eligibility, included in systematic review, and, if applicable, included in the meta-analysis). | | 4 | |
| Data collection process | | 10 | | Describe method of data extraction from reports (e.g., piloted forms, independently, in duplicate) and any processes for obtaining and confirming data from investigators. | | 4 | |
| Data items | | 11 | | List and define all variables for which data were sought (e.g., PICOS, funding sources) and any assumptions and simplifications made. | | 4 | |
| Risk of bias in individual studies | | 12 | | Describe methods used for assessing risk of bias of individual studies (including specification of whether this was done at the study or outcome level), and how this information is to be used in any data synthesis. | | 4 | |
| Summary measures | | 13 | | State the principal summary measures (e.g., risk ratio, difference in means). | | 4 | |
| Synthesis of results | | 14 | | Describe the methods of handling data and combining results of studies, if done, including measures of consistency (e.g., I^2^) for each meta-analysis. | | 4 | |
| Section/topic | | # | | Checklist item | | Reported on page # | |
| Risk of bias across studies | | 15 | | Specify any assessment of risk of bias that may affect the cumulative evidence (e.g., publication bias, selective reporting within studies). | | 4 | |
| Additional analyses | | 16 | | Describe methods of additional analyses (e.g., sensitivity or subgroup analyses, meta-regression), if done, indicating which were pre-specified. | | 4 | |
| **RESULTS** | | | | | |  | |
| Study selection | | 17 | | Give numbers of studies screened, assessed for eligibility, and included in the review, with reasons for exclusions at each stage, ideally with a flow diagram. | | 4 | |
| Study characteristics | | 18 | | For each study, present characteristics for which data were extracted (e.g., study size, PICOS, follow-up period) and provide the citations. | | 4-5 | |
| Risk of bias within studies | | 19 | | Present data on risk of bias of each study and, if available, any outcome level assessment (see item 12). | | 5 | |
| Results of individual studies | | 20 | | For all outcomes considered (benefits or harms), present, for each study: (a) simple summary data for each intervention group (b) effect estimates and confidence intervals, ideally with a forest plot. | | 5 | |
| Synthesis of results | | 21 | | Present results of each meta-analysis done, including confidence intervals and measures of consistency. | | 5-6 | |
| Risk of bias across studies | | 22 | | Present results of any assessment of risk of bias across studies (see Item 15). | | 6 | |
| Additional analysis | | 23 | | Give results of additional analyses, if done (e.g., sensitivity or subgroup analyses, meta-regression [see Item 16]). | | 6 | |
| **DISCUSSION** | | | | | |  | |
| Summary of evidence | | 24 | | Summarize the main findings including the strength of evidence for each main outcome; consider their relevance to key groups (e.g., healthcare providers, users, and policy makers). | | 6 | |
| Limitations | | 25 | | Discuss limitations at study and outcome level (e.g., risk of bias), and at review-level (e.g., incomplete retrieval of identified research, reporting bias). | | 6-9 | |
| Conclusions | | 26 | | Provide a general interpretation of the results in the context of other evidence, and implications for future research. | | 9 | |
| **FUNDING** | | | | | |  | |
| Funding | | 27 | | Describe sources of funding for the systematic review and other support (e.g., supply of data); role of funders for the systematic review. | | 10 | |

*From:*  Moher D, Liberati A, Tetzlaff J, Altman DG, The PRISMA Group (2009). Preferred Reporting Items for Systematic Reviews and Meta-Analyses: The PRISMA Statement. PLoS Med 6(6): e1000097. doi:10.1371/journal.pmed1000097

For more information, visit: **www.prisma-statement.org**.

**Table S2:** Search strategy
**PubMed database**

| Search Terms | Search Options |
| --- | --- |
| #1 | Body mass index |
| #2 | Obesity |
| #3 | Overweight |
| #4 | Fat |
| #5 | Atrial fibrillation |
| #6 | Auricular fibrillation |
| #7 | ablation |
| #8 | catheter ablation |
| #9 | pulmonary vein isolation |
| #10 | radiofrequency ablation |
| #11 | electric catheter ablation |
| #12 | #1 OR #2 OR #3 OR #4 |
| #13 | #5 OR #6 |
| #14 | #7 AND #8 AND #9 AND #10 AND #11 |
| #15 | #12 AND #13 AND #14 |

**Embase database**

| Search Terms | Search Options |
| --- | --- |
| #1 | Body mass index |
| #2 | Obesity |
| #3 | Overweight |
| #4 | Fat |
| #5 | Atrial fibrillation |
| #6 | Auricular fibrillation |
| #7 | ablation |
| #8 | catheter ablation |
| #9 | pulmonary vein isolation |
| #10 | radiofrequency ablation |
| #11 | electric catheter ablation |
| #12 | #1 OR #2 OR #3 OR #4 |
| #13 | #5 OR #6 |
| #14 | #7 AND #8 AND #9 AND #10 AND #11 |
| #15 | #12 AND #13 AND #14 |

**Cochrane library**

| Search Terms | Search Options |
| --- | --- |
| #1 | Body mass index |
| #2 | Obesity |
| #3 | Overweight |
| #4 | Fat |
| #5 | Atrial fibrillation |
| #6 | Auricular fibrillation |
| #7 | ablation |
| #8 | catheter ablation |
| #9 | pulmonary vein isolation |
| #10 | radiofrequency ablation |
| #11 | electric catheter ablation |
| #12 | #1 OR #2 OR #3 OR #4 |
| #13 | #5 OR #6 |
| #14 | #7 AND #8 AND #9 AND #10 AND #11 |
| #15 | #12 AND #13 AND #14 |

**Table S3.** Studies excluded (n=33) with reasons

| **Studies excluded** | **Reasons** |
| --- | --- |
| Prerfellner,2004[12] | Not the target outcome: Quality of life and response to PVI |
| Lee, 2004[13] | Not the target exposure: Age, presence of multiple AF foci and recurrence AF after ablation |
| Richter,2006[14] | Do not meet the inclusion: univariate analysis |
| Mainigi, 2007[15] | Not the target exposure: Location of non-pulmonary vein triggers and recurrence AF after ablation |
| Shah,2007[16] | Do not meet the inclusion: univariate analysis |
| Tao, 2008[17] | Not the target exposure: ERAF and very late of recurrence AF after ablation |
| Tang,2008[18] | Duplicated data from same population with Tang 2009 |
| Cha,2008[19] | Without target data: did not provide the RR and 95%CI |
| Wokhlu, 2010[20] | Not the target outcome: Long-Term Quality of life and recurrence AF after ablation |
| Hwang,2010[21] | Do not meet the inclusion: univariate analysis |
| Winkle,2011[22] | Duplicated data from same population |
| Mohanty,2011[23] | Not the target outcome: Long-Term Quality of life |
| Okumura, 2011[24] | Not the target exposure: Biomarkers of Inflammation and recurrence AF after ablation |
| Wong,2011[25] | Case-control study |
| Chao, 2012[26] | Not the target exposure: Left atrial diameter and recurrence AF after ablation |
| Hernandez, 2013[27] | This is a meta-analysis |
| Letsas,2013[28] | Without target data: did not provide the RR and 95%CI |
| Zhuang, 2013[29] | This is a meta-analysis |
| Pu**¨**rerfellner**,**2013[30] | Without target exposure: did not report the BMI |
| Takigawa,2014[31] | Do not meet the inclusion: univariate analysis |
| Kim,2014[32] | Do not meet the inclusion: univariate analysis |
| Baek,2016[33] | Case-control studies |
| Zylla, 2016[34] | Not the target exposure: Sex-related outcome and recurrence AF after ablation |
| Mesquita,2018[35] | Do not meet the inclusion: univariate analysis |
| Providência, 2019[36] | Duplicated data from same population |
| Nakatani,2019[37] | Do not meet the inclusion: univariate analysis |
| Shang,2019[38] | Without target exposure: did not report the BMI |
| Ciuffo,2019[39] | Without target exposure: did not report the BMI |
| Platek,2020[40] | Do not meet the inclusion: univariate analysis |
| Weinmann,2020[41] | Do not meet the inclusion: univariate analysis |
| Yu,2020[42] | Without target exposure: did not report the BMI |
| Wang, 2021[43] | Without target population: surgical ablation of valvular atrial fibrillation |
| Budzianowski,2021[44] | Without target exposure: did not report the BMI |

[1] L. Guijian, Y. Jinchuan, D. Rongzeng, Q. Jun, W. Jun, and Z. Wenqing, Impact of body mass index on atrial fibrillation recurrence: a meta-analysis of observational studies. Pacing Clin Electrophysiol 36 (2013) 748-56.

[2] C.X. Wong, T. Sullivan, M.T. Sun, R. Mahajan, R.K. Pathak, M. Middeldorp, D. Twomey, A.N. Ganesan, G. Rangnekar, K.C. Roberts-Thomson, D.H. Lau, and P. Sanders, Obesity and the Risk of Incident, Post-Operative, and Post-Ablation Atrial Fibrillation: A Meta-Analysis of 626,603 Individuals in 51 Studies. JACC Clin Electrophysiol 1 (2015) 139-152.

[3] J. Zhuang, Y. Lu, K. Tang, W. Peng, and Y. Xu, Influence of Body Mass Index on Recurrence and Quality of Life in Atrial Fibrillation Patients After Catheter Ablation: A Meta-Analysis and Systematic Review. Clinical Cardiology 36 (2013) 269-275.

[4] M.L. Mcpheeters, Newcastle-Ottawa Quality Assessment Scale. (2012).

[5] D. Aune, A. Sen, M. Prasad, T. Norat, I. Janszky, S. Tonstad, P. Romundstad, and L.J. Vatten, BMI and all cause mortality: systematic review and non-linear dose-response meta-analysis of 230 cohort studies with 3.74 million deaths among 30.3 million participants. BMJ 353 (2016) i2156.

[6] S. Greenland, and M.P. Longnecker, Methods for trend estimation from summarized dose-response data, with applications to meta-analysis. American Journal of Epidemiology 135 (1992) 1301-9.

[7] A. Abou El Khair, Recurrenceof atrial fibrillationafter catheter ablationin overweight patients, 2021.

[8] C. Xu, and D. Sar, The robust error meta-regression method for dose-response meta-analysis. International journal of evidence-based healthcare 16 (2017) 138.

[9] J. Hamling, P. Lee, R. Weitkunat, and M. Ambuhl, Facilitating meta-analyses by deriving relative effect and precision estimates for alternative comparisons from a set of estimates presented by exposure level or disease category. Stat Med 27 (2008) 954-70.

[10] L. xiao, M. jianyong, H. lin, Z. wengen, Y. ping, W. rong, and H. kui, Fluoroquinolones increase the risk of serious arrhythmias: A systematic review and meta-analysis. Medicine (Baltimore) 96 (2017) e8273.

[11] W. Zhu, R. Wan, F. Liu, J. Hu, L. Huang, J. Li, and K. Hong, Relation of Body Mass Index With Adverse Outcomes Among Patients With Atrial Fibrillation: A Meta-Analysis and Systematic Review. J Am Heart Assoc 5 (2016).

[12] H. Purerfellner, M. Martinek, J. Aichinger, H.J. Nesser, K. Kempen, and J.P. Janssen, Quality of life restored to normal in patients with atrial fibrillation after pulmonary vein ostial isolation. Am Heart J 148 (2004) 318-25.

[13] S.H. Lee, C.T. Tai, M.H. Hsieh, C.F. Tsai, Y.K. Lin, H.M. Tsao, W.C. Yu, J.L. Huang, K.C. Ueng, J.J. Cheng, Y.A. Ding, and S.A. Chen, Predictors of early and late recurrence of atrial fibrillation after catheter ablation of paroxysmal atrial fibrillation. J Interv Card Electrophysiol 10 (2004) 221-6.

[14] B. Richter, M. Gwechenberger, P. Filzmoser, M. Marx, P. Lercher, and H.D. Gossinger, Is inducibility of atrial fibrillation after radio frequency ablation really a relevant prognostic factor? Eur Heart J 27 (2006) 2553-9.

[15] S.K. Mainigi, W.H. Sauer, J.M. Cooper, S. Dixit, E.P. Gerstenfeld, D.J. Callans, A.M. Russo, R.J. Verdino, D. Lin, E.S. Zado, and F.E. Marchlinski, Incidence and predictors of very late recurrence of atrial fibrillation after ablation. J Cardiovasc Electrophysiol 18 (2007) 69-74.

[16] A.N. Shah, S. Mittal, T.C. Sichrovsky, D. Cotiga, A. Arshad, K. Maleki, W.J. Pierce, and J.S. Steinberg, Long-term outcome following successful pulmonary vein isolation: pattern and prediction of very late recurrence. J Cardiovasc Electrophysiol 19 (2008) 661-7.

[17] H. Tao, X. Liu, J. Dong, D. Long, R. Tang, B. Zheng, J. Kang, R. Yu, Y. Tian, and C. Ma, Predictors of very late recurrence of atrial fibrillation after circumferential pulmonary vein ablation. Clin Cardiol 31 (2008) 463-8.

[18] R.-B. Tang, J.-Z. Dong, X.-P. Liu, J.-P. Kang, S.-F. Ding, L. Wang, D.-Y. Long, R.-H. Yu, X.-H. Liu, and S. Liu, Obstructive sleep apnoea risk profile and the risk of recurrence of atrial fibrillation after catheter ablation. Europace 11 (2008) 100-105.

[19] Y.M. Cha, P.A. Friedman, S.J. Asirvatham, W.K. Shen, T.M. Munger, R.F. Rea, P.A. Brady, A. Jahangir, K.H. Monahan, D.O. Hodge, R.A. Meverden, B.J. Gersh, S.C. Hammill, and D.L. Packer, Catheter ablation for atrial fibrillation in patients with obesity. Circulation 117 (2008) 2583-90.

[20] A. Wokhlu, K.H. Monahan, D.O. Hodge, S.J. Asirvatham, P.A. Friedman, T.M. Munger, D.J. Bradley, C.M. Bluhm, J.M. Haroldson, and D.L. Packer, Long-term quality of life after ablation of atrial fibrillation the impact of recurrence, symptom relief, and placebo effect. J Am Coll Cardiol 55 (2010) 2308-16.

[21] H. Jin Hwang, J. Myung Lee, B. Joung, B.H. Lee, J.B. Kim, M.H. Lee, Y. Jang, and S.S. Kim, Atrial electroanatomical remodeling as a determinant of different outcomes between two current ablation strategies: circumferential pulmonary vein isolation vs pulmonary vein isolation. Clin Cardiol 33 (2010) E69-74.

[22] R.A. Winkle, R.H. Mead, G. Engel, and R.A. Patrawala, Relation of early termination of persistent atrial fibrillation by cardioversion or drugs to ablation outcomes. The American journal of cardiology 108 (2011) 374-379.

[23] S. Mohanty, P. Mohanty, L. Di Biase, R. Bai, A. Dixon, D. Burkhardt, J.G. Gallinghouse, R. Horton, J.E. Sanchez, S. Bailey, J. Zagrodzky, and A. Natale, Influence of body mass index on quality of life in atrial fibrillation patients undergoing catheter ablation. Heart Rhythm 8 (2011) 1847-52.

[24] Y. Okumura, I. Watanabe, T. Nakai, K. Ohkubo, T. Kofune, M. Kofune, K. Nagashima, H. Mano, K. Sonoda, Y. Kasamaki, and A. Hirayama, Impact of biomarkers of inflammation and extracellular matrix turnover on the outcome of atrial fibrillation ablation: importance of matrix metalloproteinase-2 as a predictor of atrial fibrillation recurrence. J Cardiovasc Electrophysiol 22 (2011) 987-93.

[25] C.X. Wong, H.S. Abed, P. Molaee, A.J. Nelson, A.G. Brooks, G. Sharma, D.P. Leong, D.H. Lau, M.E. Middeldorp, K.C. Roberts-Thomson, G.A. Wittert, W.P. Abhayaratna, S.G. Worthley, and P. Sanders, Pericardial fat is associated with atrial fibrillation severity and ablation outcome. J Am Coll Cardiol 57 (2011) 1745-51.

[26] T.F. Chao, H.M. Tsao, Y.J. Lin, C.F. Tsai, W.S. Lin, S.L. Chang, L.W. Lo, Y.F. Hu, T.C. Tuan, K. Suenari, C.H. Li, B. Hartono, H.Y. Chang, K. Ambrose, T.J. Wu, and S.A. Chen, Clinical outcome of catheter ablation in patients with nonparoxysmal atrial fibrillation: results of 3-year follow-up. Circ Arrhythm Electrophysiol 5 (2012) 514-20.

[27] A.V. Hernandez, R. Kaw, V. Pasupuleti, P. Bina, J.P. Ioannidis, H. Bueno, E. Boersma, M. Gillinov, and G. Cardiovascular Meta-Analyses Research, Association between obesity and postoperative atrial fibrillation in patients undergoing cardiac operations: a systematic review and meta-analysis. Ann Thorac Surg 96 (2013) 1104-16.

[28] K.P. Letsas, C.H. Siklody, P. Korantzopoulos, R. Weber, G. Burkle, C.C. Mihas, D. Kalusche, and T. Arentz, The impact of body mass index on the efficacy and safety of catheter ablation of atrial fibrillation. Int J Cardiol 164 (2013) 94-8.

[29] J. Zhuang, Y. Lu, K. Tang, W. Peng, and Y. Xu, Influence of body mass index on recurrence and quality of life in atrial fibrillation patients after catheter ablation: a meta-analysis and systematic review. Clin Cardiol 36 (2013) 269-75.

[30] Y. Sotomi, K. Inoue, N. Ito, R. Kimura, Y. Toyoshima, M. Masuda, A. Doi, K. Iwakura, A. Okamura, Y. Koyama, M. Date, and K. Fujii, Cause of very late recurrence of atrial fibrillation or flutter after catheter ablation for atrial fibrillation. Am J Cardiol 111 (2013) 552-6.

[31] M. Takigawa, A. Takahashi, T. Kuwahara, K. Okubo, Y. Takahashi, Y. Watari, K. Takagi, T. Fujino, S. Kimura, H. Hikita, M. Tomita, K. Hirao, and M. Isobe, Long-term follow-up after catheter ablation of paroxysmal atrial fibrillation: the incidence of recurrence and progression of atrial fibrillation. Circ Arrhythm Electrophysiol 7 (2014) 267-73.

[32] T.H. Kim, J. Park, J.K. Park, J.S. Uhm, B. Joung, M.H. Lee, and H.N. Pak, Pericardial fat volume is associated with clinical recurrence after catheter ablation for persistent atrial fibrillation, but not paroxysmal atrial fibrillation: an analysis of over 600-patients. Int J Cardiol 176 (2014) 841-6.

[33] Y.S. Baek, P.S. Yang, T.H. Kim, J.S. Uhm, J.Y. Kim, B. Joung, M.H. Lee, and H.N. Pak, Delayed recurrence of atrial fibrillation 2years after catheter ablation is associated with metabolic syndrome. Int J Cardiol 223 (2016) 276-281.

[34] M.M. Zylla, J. Brachmann, T. Lewalter, E. Hoffmann, K.H. Kuck, D. Andresen, S. Willems, L. Eckardt, J. Tebbenjohanns, S.G. Spitzer, B. Schumacher, M. Hochadel, J. Senges, H.A. Katus, and D. Thomas, Sex-related outcome of atrial fibrillation ablation: Insights from the German Ablation Registry. Heart Rhythm 13 (2016) 1837-44.

[35] J. Mesquita, A.M. Ferreira, D. Cavaco, P. Carmo, M. Madeira, P. Freitas, F.M. Costa, F. Morgado, M. Mendes, and P. Adragao, Impact of prophylactic cavotricuspid isthmus ablation in atrial fibrillation recurrence after a first pulmonary vein isolation procedure. Int J Cardiol 259 (2018) 82-87.

[36] R. Providencia, C. de Asmundis, J. Chun, G. Chierchia, P. Defaye, F. Anselme, A. Creta, P.D. Lambiase, B. Schmidt, S. Chen, R.J. Hunter, S. Combes, S. Honarbakhsh, N. Combes, M.J. Sousa, Z. Jebberi, J.P. Albenque, and S. Boveda, Catheter ablation of atrial fibrillation in patients with heart failure with reduced ejection fraction: Real world experience from six European centers. J Cardiovasc Electrophysiol 30 (2019) 1270-1277.

[37] Y. Nakatani, T. Sakamoto, Y. Yamaguchi, Y. Tsujino, N. Kataoka, K. Nishida, K. Mizumaki, and K. Kinugawa, Impacts of the body size on the left atrial wall thickness and atrial fibrillation recurrence after catheter ablation. Heart Vessels 34 (2019) 1351-1359.

[38] L. Shang, M. Shao, Q. Guo, J. Xiaokereti, Y. Zhao, Y. Lu, L. Zhang, B. Tang, and X. Zhou, Association of Obesity Measures with Atrial Fibrillation Recurrence After Cryoablation in Patients with Paroxysmal Atrial Fibrillation. Med Sci Monit 26 (2020) e920429.

[39] L. Ciuffo, H. Nguyen, M.D. Marques, K.N. Aronis, B. Sivasambu, H.D. de Vasconcelos, S. Tao, D.D. Spragg, J.E. Marine, R.D. Berger, J.A.C. Lima, H. Calkins, and H. Ashikaga, Periatrial Fat Quality Predicts Atrial Fibrillation Ablation Outcome. Circ Cardiovasc Imaging 12 (2019) e008764.

[40] A.E. Platek, A. Szymanska, I. Kalaszczynska, F.M. Szymanski, J. Sierdzinski, and K.J. Filipiak, Usefulness of Visfatin as a Predictor of Atrial Fibrillation Recurrence After Ablation Procedure. Am J Cardiol 125 (2020) 415-419.

[41] K. Weinmann, C. Bothner, M. Rattka, D. Aktolga, Y. Teumer, W. Rottbauer, T. Dahme, and A. Pott, Pulmonary vein isolation with the cryoballoon in obese atrial fibrillation patients - Does weight have an impact on procedural parameters and clinical outcome? Int J Cardiol 316 (2020) 137-142.

[42] H.T. Yu, I.S. Kim, T.H. Kim, J.S. Uhm, J.Y. Kim, B. Joung, M.H. Lee, and H.N. Pak, Persistent atrial fibrillation over 3 years is associated with higher recurrence after catheter ablation. J Cardiovasc Electrophysiol 31 (2020) 457-464.

[43] D.-J. Wang, and Y. Wang, A novel predictive model of recurrence after surgical ablation of valvular atrial fibrillation. Available at SSRN 3948432 (2021).

[44] J. Budzianowski, J. Hiczkiewicz, K. Łojewska, E. Kawka, R. Rutkowski, and K. Korybalska, Predictors of Early-Recurrence Atrial Fibrillation after Catheter Ablation in Women and Men with Abnormal Body Weight. Journal of Clinical Medicine 10 (2021) 2694.

**Table S4**. Quality assessment of included studies

| Author  (Publication Year) | Newcastle-Ottawa Scale | | | | | | | | | |
| --- | --- | --- | --- | --- | --- | --- | --- | --- | --- | --- |
|  | Selection | | | Comparability | | | Outcome | | | Total |
|  | a | b | c | d | e | f | g | h | i |  |
| Jongnarangsin et al,2008 | 1 | 1 | 1 | 1 | 1 | 1 | 1 | 1 | 0 | 8 |
| Chang et al,2008 | 0 | 1 | 1 | 1 | 0 | 1 | 1 | 1 | 0 | 6 |
| Letsas et al,2009 | 1 | 1 | 1 | 1 | 1 | 1 | 1 | 1 | 0 | 8 |
| Tang et al,2009 | 1 | 1 | 1 | 1 | 1 | 1 | 1 | 1 | 1 | 9 |
| Patel et al, 2010, | 1 | 1 | 1 | 1 | 0 | 1 | 1 | 1 | 0 | 7 |
| Chilukuri et al, 2010 | 1 | 1 | 1 | 1 | 0 | 1 | 1 | 0 | 1 | 7 |
| Kang et al, 2012 | 0 | 1 | 1 | 1 | 1 | 1 | 1 | 0 | 0 | 6 |
| Cai et al,2011 | 1 | 1 | 1 | 1 | 0 | 1 | 1 | 1 | 0 | 8 |
| He et al,2013 | 1 | 1 | 1 | 1 | 0 | 1 | 1 | 1 | 0 | 7 |
| Sotomi et al,2013 | 1 | 1 | 1 | 1 | 0 | 1 | 1 | 1 | 0 | 7 |
| Bake et al, 2016 | 1 | 1 | 1 | 1 | 1 | 1 | 1 | 1 | 1 | 9 |
| Bunch et al, 2016 | 1 | 1 | 1 | 1 | 1 | 1 | 1 | 1 | 0 | 8 |
| Winkle et al, 2017 | 1 | 1 | 1 | 1 | 1 | 1 | 1 | 1 | 0 | 8 |
| Sivasambu et al,2017 | 1 | 1 | 1 | 1 | 0 | 1 | 1 | 0 | 0 | 6 |
| Deng et al,2018 | 1 | 1 | 1 | 1 | 1 | 1 | 1 | 1 | 0 | 8 |
| Maat et al, 201 | 1 | 1 | 1 | 1 | 0 | 1 | 1 | 1 | 1 | 8 |
| Glover et al,2018 | 1 | 1 | 1 | 1 | 0 | 1 | 1 | 1 | 0 | 7 |
| Providência et al, 2019 | 1 | 1 | 1 | 1 | 1 | 1 | 1 | 1 | 0 | 8 |
| Trines et al,2019 | 1 | 1 | 1 | 1 | 0 | 1 | 1 | 1 | 0 | 7 |
| Baek et al, 2020 | 1 | 1 | 1 | 1 | 1 | 1 | 1 | 1 | 1 | 9 |
| Nunez et al,2020 | 1 | 1 | 1 | 1 | 0 | 1 | 1 | 1 | 0 | 7 |
| Bose et al, 2020, | 0 | 1 | 1 | 1 | 1 | 1 | 1 | 1 | 0 | 7 |
| Kong et al, 2020 | 1 | 1 | 1 | 1 | 1 | 1 | 1 | 1 | 1 | 9 |
| Donnellan et al, 2020 | 1 | 1 | 1 | 1 | 0 | 1 | 1 | 1 | 0 | 7 |
| Khair et al,2021 | 1 | 1 | 1 | 1 | 1 | 1 | 1 | 0 | 0 | 7 |
| Mugnai et al, 2021 | 1 | 1 | 1 | 1 | 0 | 1 | 1 | 0 | 1 | 7 |

1. Representativeness of the exposed cohort.
2. Selection of the non-exposed cohort.
3. Ascertainment of exposure.
4. Demonstration that outcome of interest was not present at start of study.
5. Comparability of cohorts on the basis of the design or analysis (adjusted for age).
6. Comparability of cohorts on the basis of the design or analysis (adjusted for any other factor).
7. Assessment of outcome.
8. Was follow-up long enough for outcomes to occur. (>1 years).
9. Adequacy of follow-up of cohorts.

**Supplementary Table S5.** Table of RRs and 95% CIs from nonlinear dose-response analysis of BMI and AF recurrence after radiofrequency ablation

|  | AF recurrence |
| --- | --- |
| BMI | RR (95% CI) |
| 19 | 1 |
| 25 | 1.08 (0.96-1.23) |
| 30 | 1.38 (1.18-1.63) |
| 35 | 1.95 (1.41-2.74) |
| 40 | 2.78(1.67-4.66) |

**SUPPLEMENTAL FIGURES**

**A B**


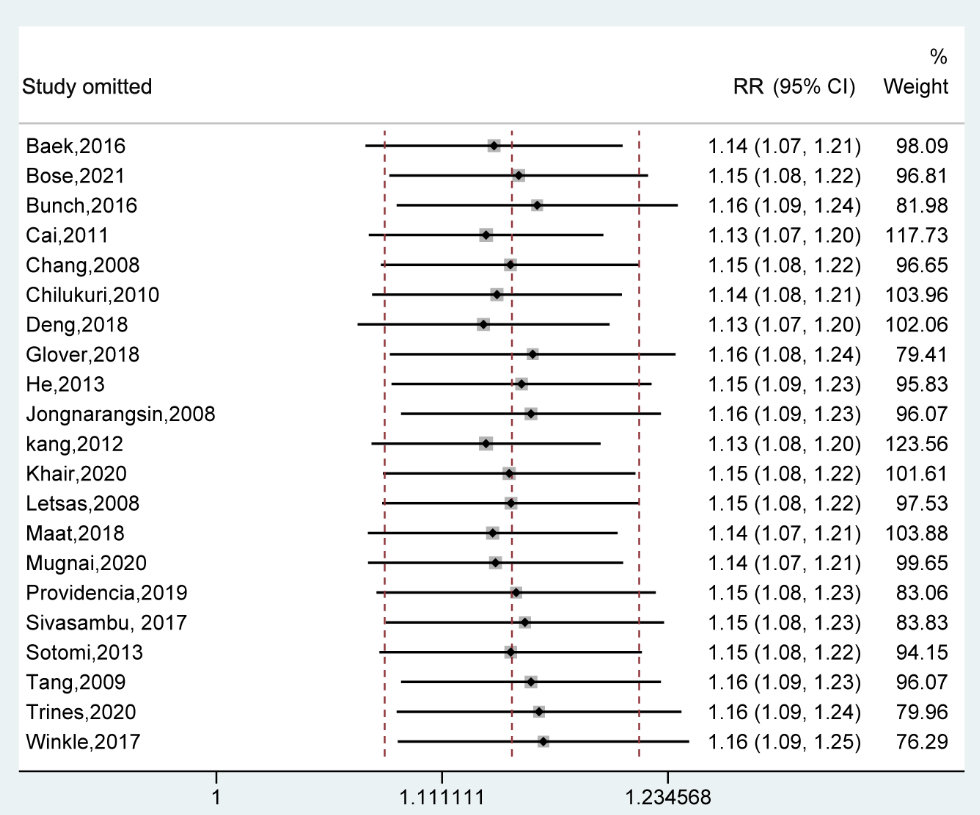

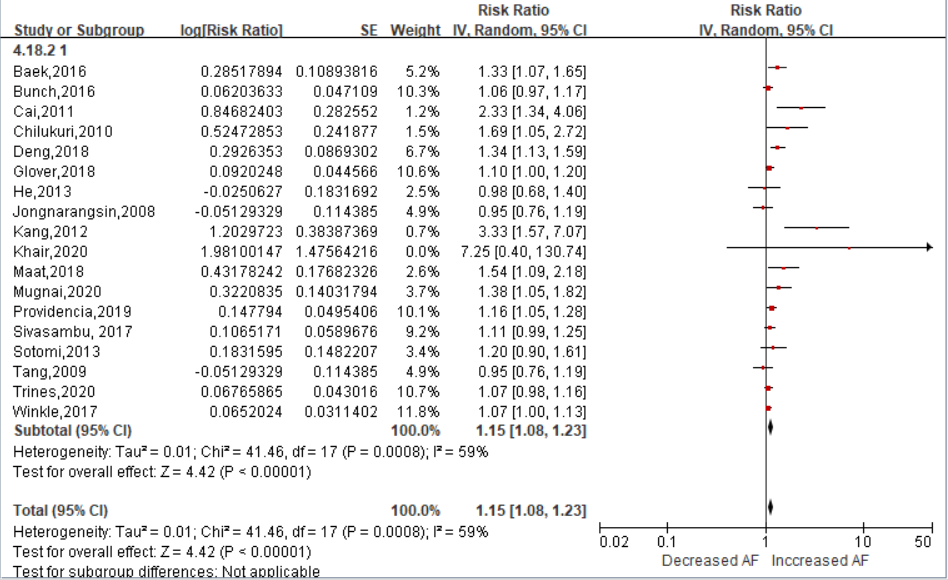


**Supplemental Figure S1. Results of leave-one-out method (A) and excluding studies with a blank period<3 months (B) in s**[**ensitivity**](javascript:;) [**analysis**](javascript:;) **of body mass index and atrial fibrillation recurrence in patients undergoing radiofrequency ablation, exposure-effect analysis, per 5 unit.** The three vertical dotted lines denote the pooled random effect of risk ratio and 95% CI (risk ratio = 1.15, 95% CI 1.08-1.22). The horizontal lines and dot the indicate the risk ratio and 95% CIs applying the leave-one-out method.


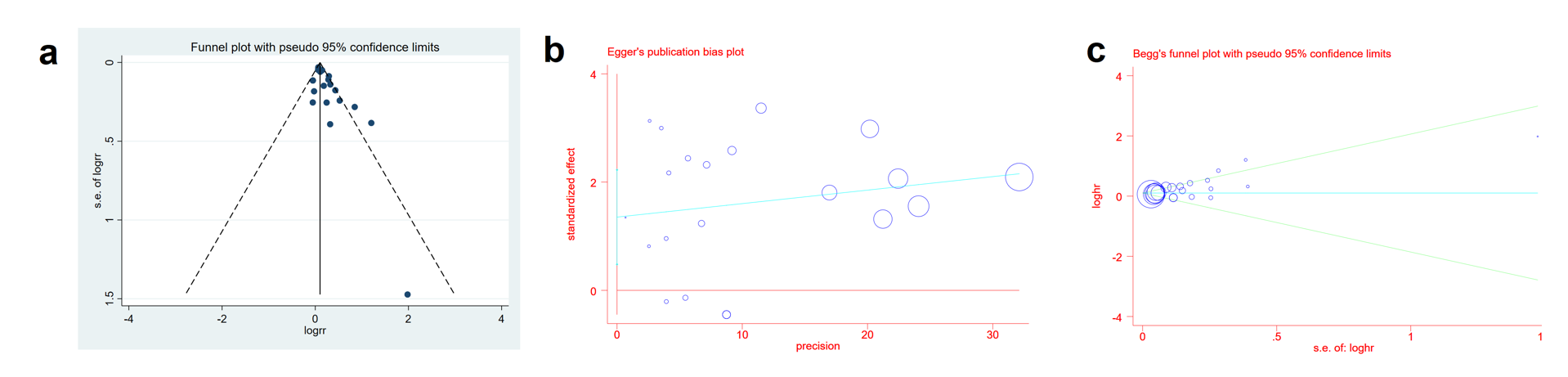


**Supplemental Figure S2: Publication bias test of the body mass index and atrial fibrillation recurrence in patients undergoing radiofrequency ablation** **in the results of exposure-effect analysis, per 5 unit. A: Funnel plot；B：Begg’s test; C: Egger’s test**


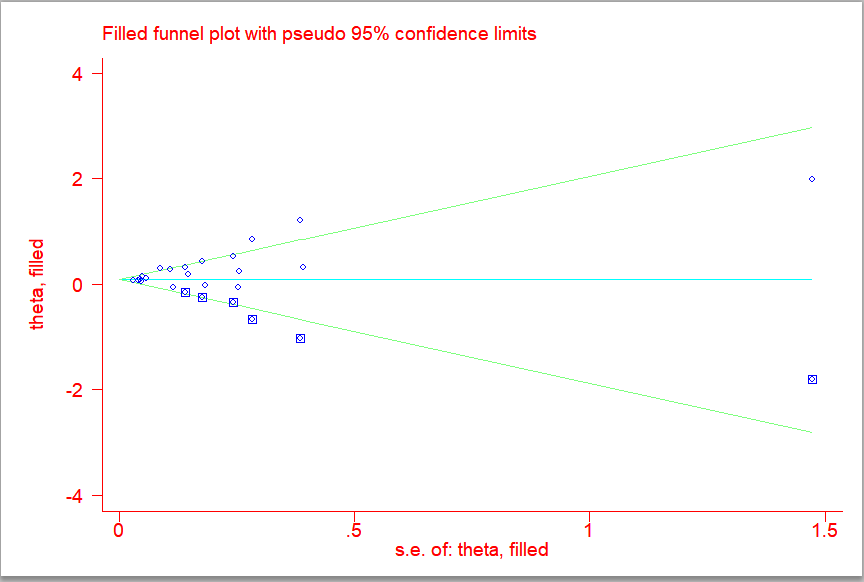


**Supplemental Figure S3: Meta-trim funnel; the "trim and fill" method was used to adjust for publication bias in funnel plot that shows symmetrical distribution of the studies**
